# Intracellular nutrient storage during ice algal spring blooms in the Canadian high Arctic

**DOI:** 10.1016/j.isci.2025.113148

**Published:** 2025-07-18

**Authors:** C.J. Mundy, Eva Leu, Karley Campbell, Virginie Galindo, Maurice Levasseur, Michel Poulin, Jean-Éric Tremblay, Michel Gosselin

**Affiliations:** 1 Centre for Earth Observation Science (CEOS), Clayton H. Riddell Faculty of Environment, Earth, and Resources, University of Manitoba, Winnipeg, MB, Canada; 2 Akvaplan-Niva AS, Fram Centre, 9296 Tromsø, Norway; 3 Department of Arctic and Marine Biology, UiT the Arctic University of Norway, Tromsø, Norway; 4 Direction générale de la conservation de la biodiversité, Ministère de l’Environnement et de la Lutte contre les changements climatiques, de la Faune et des Parcs, Quebec city, QC, Canada; 5 Québec-Océan, Takuvik and Département de biologie, Université Laval, Quebec City, QC, Canada; 6 Research and Collections, Canadian Museum of Nature, Ottawa, ON, Canada; 7 Institut des sciences de la mer, Université du Québec à Rimouski, Rimouski, QC, Canada

**Keywords:** marine organism, marine processes, aquatic science, aquatic biology

## Abstract

Nutrient availability influences maximum biomass, speciation, cellular composition, and overall phenology of Arctic spring ice algal blooms. However, how ice algae obtain nutrients from their environment is not well understood. Previously documented positive relationships between sea ice nutrient concentrations and algal biomass implied that ice algae maintain an intracellular nutrient pool. Here, we provide direct evidence that sea ice diatoms store intracellular nitrate + nitrite and silicic acid well above that available in their ambient environment. Differential retention of intracellular pools released during standard melt processing techniques led to an increase in the apparent dissolved N:Si ratio measured in ice melt samples that likely influenced interpretations of Si-limitation in some previous studies. It is hypothesized that the ability of ice algae to store intracellular nutrient reserves represents a beneficial adaptation for ice algae to extend blooms under a periodic tidal-pulsed flux of nutrients to the ice bottom environment.

## Highlights

• Ice algae internally store nutrients at levels exceeding those in their environment
• Differential retention of intracellular pools can impact bulk ice melt measurements
• Intracellular nutrients help extend ice algal blooms under a pulsed nutrient supply

## Graphical abstract

**Figure:**
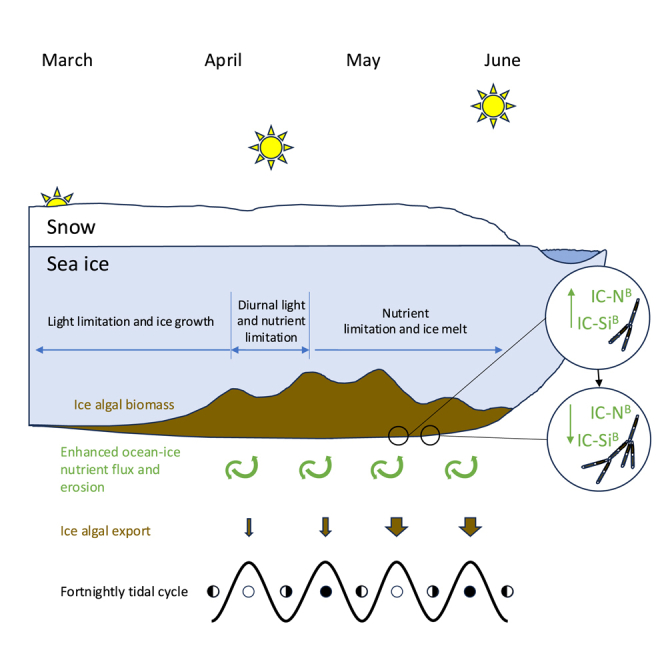

## Introduction

Ice algae account for a critical pulse of primary production in Arctic seas, helping to support a lipid-rich food web with typically strong ice-pelagic-benthic coupling and accounting for an important carbon export to the benthos.^1^^,^^2^^,^^3^ With a warming climate, sea ice is becoming thinner, as old ice is replaced by younger first-year ice, and melting earlier and for a longer period,^4^ while snow depth on sea ice is decreasing.^5^ These changes can have both positive (e.g., more light earlier in the season) and negative (shortened growth season due to ice loss) implications on ice algal production. However, it is access to nutrients that mainly influences total production, taxonomic composition, and potentially termination of the spring ice algal bloom in ice-covered seas.^6^^,^^7^ Nutrient access can also greatly influence algal biomass composition,^8^^,^^9^ and thus the quality of food available to the Arctic food web.^10^

A detailed examination of ice algal nutrient dynamics requires the ability to accurately measure nutrient concentrations that are available for algal growth and biomass accumulation. This is not a straightforward task with sea ice, where nutrient ions are concentrated in the liquid brine environment and regularly exchanged with surface ocean waters.^11^^,^^12^ Nutrient measurements for ice algal studies can include measurements from sack-hole brine collection,^13^ ice-water interface/surface ocean samples,^14^^,^^15^ and pure ice melt (hereinafter referred to as bulk ice).^16^ All methods have their limitations. With sack-hole collection, it is not directly apparent where brine is collected from,^17^^,^^18^ and thus whether it can be representative of the actual ice algal environment. Wintertime surface waters can provide a good proxy of nutrient availability, but again it is not truly representative of availability in the immediate ice algal environment during the growth period.

Bulk ice core sections can zero-in on the ice algal environment; however, dilution of salts during melt processing of the sample can cause osmotic-induced stress on cells.^19^ During the ice algal spring bloom, the bottom-ice environment (e.g., bottommost 5 cm of the ice cover) can range in temperature from approximately −1.8°C (freezing) to −3°C, which corresponds to brine salinities of 32.2 to 52.5.^20^ In contrast, bulk ice melt salinities are typically <12. Therefore, bulk ice melt processing techniques rapidly expose ice algal cells to a hypotonic environment, leading to the flow of water into cells through osmosis, causing cells to swell and potentially burst. Furthermore, the melt of ice crystals significantly dilutes ambient nutrient concentrations.^21^ The resultant stress on the ice algal cells was shown to significantly impact photophysiology, primary production,^22^ chlorophyll *a* concentration,^23^ and cell count^19^ estimates. Accordingly, positive relationships between nutrient concentrations measured in bulk ice melt samples and algal biomass led to the hypothesis that algae maintain an intracellular (IC) nutrient pool that is released upon melt processing.^16^ However, to the authors’ knowledge, there is only a single direct documentation of IC nutrient pools in sea ice algae,^24^ yet IC nutrient storage is well-documented in pennate diatoms,^25^ the dominant taxa of the bottom-ice algal spring bloom.^26^

Algae have long been known to store IC nutrient pools (e.g., ^27^^,^^28^^,^^29^). In freshwater and marine diatoms, IC nutrient pools can be significant, since the fraction of their protoplast volume occupied by vacuoles varies between 0.45 and 0.90.^30^ For example, diatoms have the capacity to take up silicic acid from their environment and store it at concentrations that can exceed saturation limits.^31^ This capacity is likely assisted by binding organic molecules within the cell that stabilize the soluble silicon; however, the exact mechanism of intracellular storage and transport of silicic acid is still not well understood.^32^ Recently, Stief et al.^25^ showed that the intracellular nitrate storage by pelagic and benthic diatoms can be an important nitrogen pool in freshwater and marine ecosystems. Research in benthic environments has highlighted the ability of pennate diatoms, in particular, to uptake and store nitrate intracellularly up to concentrations of 274 mmol L^−1^, orders of magnitude greater than that available in their natural environment.^33^ The storage of IC-nitrate was shown to either support assimilatory nitrate reduction for the build-up of cellular biomass when conditions become more favorable (e.g., light access increases) or dissimilatory reduction where nitrate acts as an electron acceptor to survive dark and anoxic conditions.^34^ Thus, IC-nitrate storage provides a highly beneficial adaptation for diatoms to survive adverse conditions from darkness to limited nutrient access to anoxic conditions, such as those experienced in the upper layers of the benthic environment.^34^^,^^35^ Arctic sea ice also represents an environment influenced by seasonal darkness, limited brine permeability, and thus access to nutrients, and even documented anoxic conditions in the lower layers of sea ice.^36^ It is noted that the possibility of IC-nitrate storage contributing to ice algal winter survival adds to already suggested strategies of facultative heterotrophy, a reduced metabolic state, and utilization of additional intracellular energy stores as well as exopolymeric substances released into the sea ice environment.^37^^,^^38^^,^^39^^,^^40^ Furthermore, new nutrient access for the ice algal community is governed via supply from the underlying water column.^11^^,^^41^^,^^42^ Shear at the ice-ocean interface, created via periodic tidal forcing in coastal landfast ice environments or drag of the ice-pack against ocean currents,^43^ can increase ocean-ice fluxes of heat, fluid, and diffusion of nutrients, across the ice-ocean boundary layer.^11^^,^^44^ The resultant pulsed supply of new nutrients can extend an ice algal bloom,^11^ where surge uptake of nutrients^45^ would represent a valuable adaptation.

As part of a process-based landfast ice study near Resolute Bay, Nunavut, called the Arctic-ice covered ecosystem (Arctic-ICE) project, a dataset was collected during the 2010 and 2012 spring ice algal bloom to test the hypothesis that ice algae maintain an IC-nutrient pool. The main objectives of this work are (1) to improve the interpretation of estimates of nutrient concentrations in bulk ice by comparing them with intracellular nutrient stores, and (2) to examine the influence of tidally induced under-ice currents on the intracellular nutrient dynamics of ice algae during a bloom. We show that intracellular nutrient storage may play a critical role in the sustained production of ice algae during their spring bloom.

## Results

### The spring bloom in resolute passage

**Figure 1:**
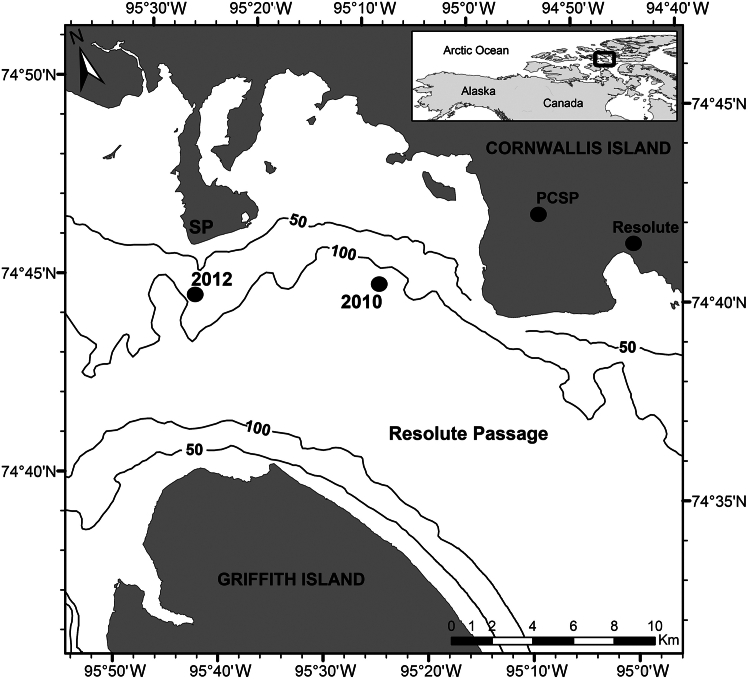
Map of the study sites Map of the 2010 and 2012 landfast first-year sea ice sites of the Arctic-ICE project. Map acronyms include polar continental shelf program (PCSP) and sheringham point (SP). Isobaths are in meters.

Site-specific details were described elsewhere for the Arctic-ICE 2010^46^ and 2012^47^ field projects (Figure 1), and are summarized in Table 1. In 2010 (8 May to 18 June), sampling extended beyond the dry snow period (until 6 June) and well into advanced spring melt with the appearance of melt ponds at “thin snow” sites on 15 June 2010. Averaged (±SD) sampled snow depth and ice thickness prior to snow melt onset (11 May to 6 June) were 15.4 ± 3.3 and 141 ± 2 cm, respectively. Melt pond depths reached 4.3 ± 1.3 cm with a corresponding ice thickness of 129 ± 3 cm on 18 June at “low snow” sites. In contrast, “thick snow” sites decreased in snow depth to 8.8 ± 1.3 cm while ice thickness remained relatively constant at 144 ± 3 cm by 18 June.^46^ The 2012 dataset (19 May to 8 June) was collected from snow-covered sea ice, ending just before the formation of melt ponds, with averaged sampled snow depth and ice thickness of 11.8 ± 2.1 and 125 ± 1 cm, respectively, prior to melt onset (31 May). Thereafter, snow depth and ice thickness decreased to average 9.0 ± 6.1 and 117 ± 1 cm, respectively, between 4 and 8 June due to air temperatures rising above 0°C.^47^ Both datasets captured the decline phase of the ice algal blooms, marked by significant decreases in bottom-ice chlorophyll *a* (chl *a*) in both 2010 (*r*^2^ = 0.77, *p* < 0.001) and 2012 (*r*^2^ = 0.99, *p* < 0.001). These blooms were largely comprised of pennate diatoms throughout the spring, accounting for 82.2 ± 3.3% and 82.1 ± 4.1% of the bottom-ice algal community cell abundance in 2010 and 2012, respectively. Furthermore, pennate diatoms contributed 98.0 ± 0.7 and 96.6 ± 0.6% to the total cell volume of the ice algal community in 2010 and 2012, respectively. The three most dominant pennate genera included *Nitzschia* (50%), *Navicula* (30%), and *Synedropsis* (7%) in 2010 and *Nitzschia* (50%), *Navicula* (32%), and *Entomoneis* (5%) in 2012. *Nitzschia frigida* and *Navicula pelagica* were the dominant species in both years, together accounting for 65% (2010) and 71% (2012) of the identified pennate diatoms.

**Table 1:** Field project summary of pertinent data

Year	2010	2012
Sampling period	8 May to 18 June	19 May to 8 June
Snow melt [pond] onset date	6 June [15 June]	31 May [NA]
Snow depth (cm)	13.4 (±4.9)	10.9 (±3.5)
Ice thickness (cm)	141 (±2)	123 (±4)
Water column NO_3_ + NO_2_ at 2-m depth (μmol L^−1^)	7.32 (±0.43)	7.67 (±0.38)
Water column Si(OH)_4_ at 2-m depth (μmol L^−1^)	14.8 (±0.58)	14.7 (±0.9)
Bulk ice NO_3_ + NO_2_ (μmol L^−1^)	NA	19.5 (±11.6)
Bulk ice Si(OH)_4_ (μmol L^−1^)	NA	11.2 (±6.4)
Chl *a*-specific intracellular NO_3_ + NO_2_ (μmol mg chl *a*^−1^)	11.7 (±5.2)	12.8 (±3.4)
Chl *a*-specific intracellular Si(OH)_4_ (μmol mg chl *a*^−1^)	34.2 (±21.8)	59.7 (±27.3)
Bottom-ice chl *a* (mg m^−2^)	20.1 (±9.6)	53.0 (±30.1)
Bottom-ice max. [min.] chl *a* (mg m^−2^)	45.8 [3.2]	99.9 [0.42]
Bottom-ice algal abundance (cells L^−1^)	1.63 (±0.74) × 10^8^	3.04 (±2.16) × 10^8^
Contribution of pennate diatoms to total cell abundance (%)	82.2 (±3.3)	82.1 (±4.1)
Bottom-ice total community cell volume (μm^3^ L^−1^)	3.05 (±1.39) × 10^11^	6.80 (±3.96) × 10^11^
Intracellular NO_3_ + NO_2_ concentration (mmol L^−1^)	19.2 (±10.6)	26.0 (±8.6)
Intracellular Si(OH)_4_ concentration (mmol L^−1^)	58.4 (±46.0)	128 (±79)
Intracellular NO_3_ + NO_2_ content (fmol cell^−1^)	36.3 (±17.2)	68.2 (±41.6)
Intracellular Si(OH)_4_ content (fmol cell^−1^)	110 (±91)	251 (±134)
Contribution of pennate diatoms to total cell volume (%)	98.0 (±0.7)	96.6 (±0.2)

A summary of pertinent physical, chemical, and biological variables observed during Arctic-ICE 2010 and 2012 field projects. Sampling period averages (±SD) are presented where appropriate. Water column nutrient averages for 2010 are only reported for the period of 8 May to 5 June (see text). NA stands for not available. Bulk ice and bottom-ice samples refer to quantitative measurements of the bottom 3 cm of the sea ice. It is noted that in the text, Chl *a*-specific intracellular NO_3_ + NO_2_ and Si(OH)_4_ are abbreviated as IC-N^B^ and IC-Si^B^, respectively.

### Under- and bottom-ice nutrient concentrations

Nutrient concentrations at a 2-m water depth (i.e., ∼0.6–0.8 m below the ice bottom) were comparable between years (Table 1). Prior to the onset of an under-ice bloom that rapidly drew down surface nutrients following 5 June,^46^ NO_3_ + NO_2_ concentrations ranged between 6.54 and 7.83 μmol L^−1^, averaging a [NO_3_ + NO_2_]:Si(OH)_4_ (hereinafter N:Si) molar ratio of 0.50 ± 0.01 in 2010. Similarly, in 2012, NO_3_ + NO_2_ concentrations ranged between 7.23 and 8.17 μmol L^−1^, averaging an N:Si molar ratio of 0.52 ± 0.01 (Table 1).

**Figure 2:**
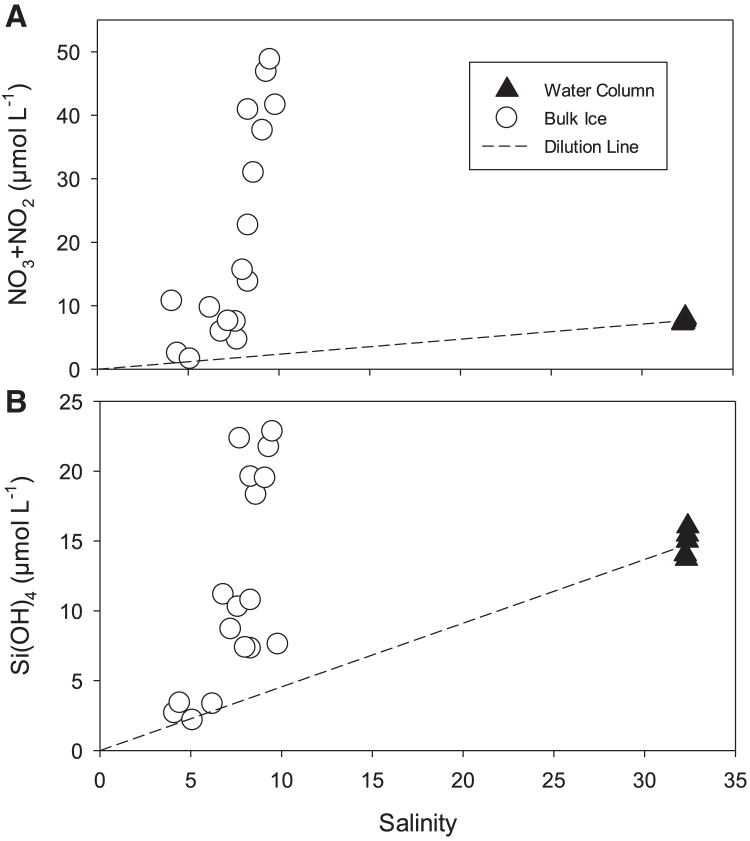
Bulk ice and surface water column nutrient concentrations versus salinity Bulk ice and 2-m water column (A) NO_3_ + NO_2_ and (B) Si(OH)_4_ concentrations as a function of salinity during 2012. Dashed lines represent dilution lines from the average 2-m water column concentration to the origin.

Bulk bottom-ice nutrients were measured only in 2012 (Table 1). Bulk ice NO_3_ + NO_2_ concentrations were on average greater in the bottom ice than that at 2-m water depth, but Si(OH)_4_ concentrations were lower. Essentially, the bulk ice N:Si molar ratio was altered within the bottom-ice environment, averaging 1.94 ± 0.67, which was significantly greater than that observed at a 2-m water depth (Kruskal-Wallis, *p* < 0.01). The difference between the under- and bottom-ice environments was even more striking when plotted against salinity of the samples, where most bulk ice nutrient concentrations fell well above the dilution line from averaged 2-m concentrations to the origin (Figure 2). Some nutrient concentrations did follow the dilution line, but only at lower bottom-ice salinities (≤6.2). Observed significant ordinary least squares (OLS) linear relationships of bulk ice nutrients (in μmol L^−1^) versus chl *a* (in μg L^−1^) were [NO_3_ + NO_2_] = 0.011[chl *a*] + 5.28 (*r*^2^ = 0.38, *p* = 0.008) and [Si(OH)_4_] = 0.005[chl *a*] + 4.21 (*r*^2^ = 0.48, *p* = 0.002).

### Ice algal intracellular nutrient concentrations

**Figure 3:**
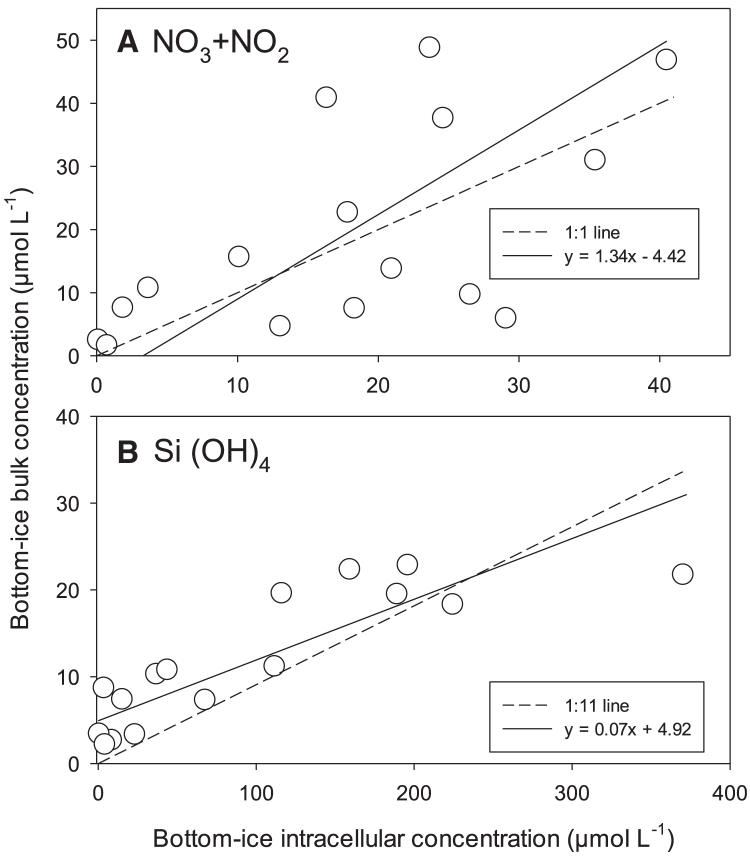
Bulk versus intracellular nutrient concentrations in the ice bottom Bottom-ice bulk nutrient concentrations versus estimates of bottom-ice intracellular nutrient concentrations of (A) NO_3_ + NO_2_ and (B) Si(OH)_4_ from 2012. Standardized major axis (SMA) regressions and constant ratios for comparison are plotted as solid and dashed lines, respectively.

Table 1 shows that intracellular (IC) nutrients were observed within the bottom-ice algal community. Chl-specific IC NO_3_ + NO_2_ (IC-N^B^) and Si(OH)_4_ (IC-Si^B^) averaged 11.7 ± 5.2 and 34.2 ± 21.7 μmol mg chl *a*^−1^ in 2010 and 12.8 ± 3.4 and 59.7 ± 27.3 μmol mg chl *a*^−1^ in 2012, respectively. These averages demonstrate that IC-Si^B^ varied more than IC-N^B^ between years. IC-N^B^ and IC-Si^B^ were multiplied by the bottom-ice chl *a* concentrations (from filtered seawater-diluted ice cores) to estimate bottom-ice IC-N and IC-Si concentration corrected to the ice melt dilution for the 2012 dataset. Bulk ice nutrient concentrations (from undiluted ice cores) were then regressed against these estimates (Figure 3). Bulk ice NO_3_ + NO_2_ concentrations fell close to the 1:1 line against bottom-ice IC-N, following a significant positive relationship (*r*^2^ = 0.37, *p* = 0.01, standardized major axis [SMA] regression). In contrast, bulk ice Si(OH)_4_ concentrations were more than an order of magnitude less than bottom-ice IC-Si estimates, falling close to a 1:11 line with a much tighter relationship (*r*^2^ = 0.72, *p* < 0.0001, SMA regression). Bottom-ice IC estimates were also calculated for the 2010 dataset, which, combined with algal cell volume and enumeration data, permitted estimates of IC concentration (per cell volume) and IC cell content (per cell), respectively (Table 1). The respective estimates for 2012, which averaged 26.0 ± 8.6 mmol L^−1^ and 68.2 ± 41.6 fmol cell^−1^ for NO_3_ + NO_2_, and 128 ± 79 mmol L^−1^ and 251 ± 134 fmol cell^−1^ for Si(OH)_4_, were approximately double that estimated for 2010. It is significant to note that the algal community averaged cell volume was greater in 2012 at 2,890 ± 930 μm^3^ cell^−1^ versus 1,890 ± 140 μm^3^ cell^−1^ in 2010.

### Temporal dynamics of intracellular nutrient concentrations

**Figure 4:**
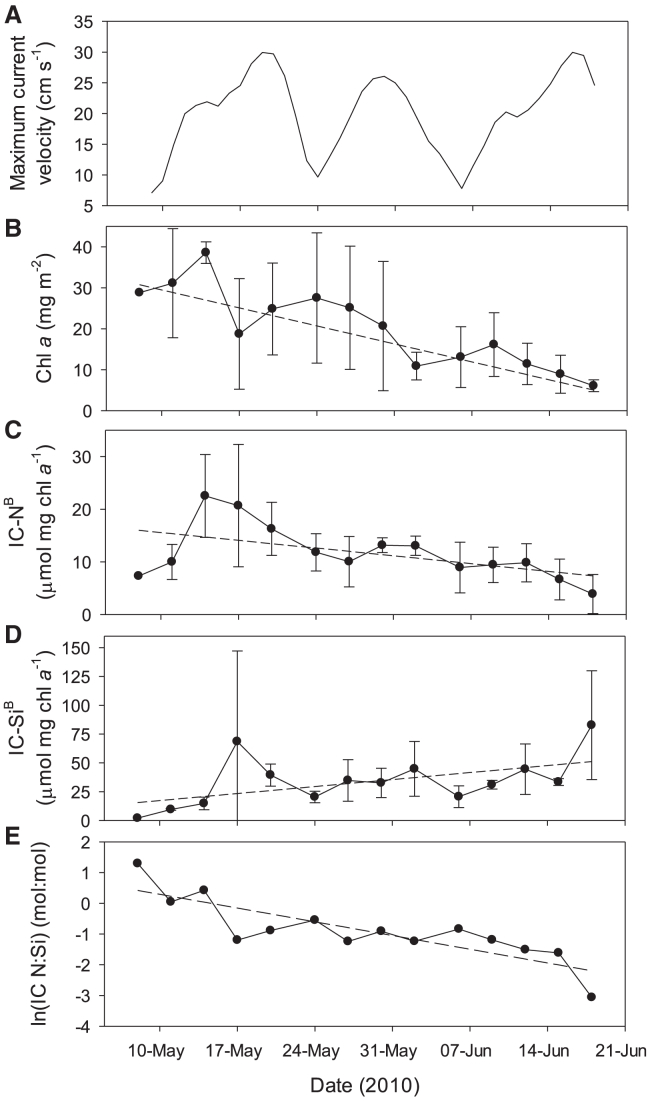
Seasonal progression of the 2010 ice algal bloom (A–E) 2010 time series of (A) interpolated maximum current velocity at a 3-m depth bin,^46^ (B) bottom-ice chlorophyll *a* concentration (chl *a*), (C) chl *a*-specific intracellular NO_3_ + NO_2_ (IC-N^B^), (D) chl *a*-specific intracellular Si(OH)_4_ (IC-Si^B^), and (E) natural log of the molar ratio of IC-N:IC-S. Error bars represent standard deviations around a calculated average from different snow depth sites. Dashed lines are significant linear ordinary least-squares regressions on the plotted averages.

The significant linear decreasing trend of bottom-ice chl *a* versus time matched that of IC-N^B^, yet was opposite to an increasing trend in IC-Si^B^ (Figure 4B–4D). These opposite temporal trends in chl-specific IC nutrients were emphasized in the N:Si intracellular nutrient ratio (mol:mol), which followed a significant exponential (natural log-linear) decay (Figure 4E). Overlain on the temporal trends were notable oscillations that corresponded to changes in daily maximum under-ice currents (Figures 4A–4D). Residuals from the linear relationships of IC-N^B^ and IC-Si^B^ against time resulted in weakly positive, significant at an α of 0.1, correlations of IC-N^B^ (*r* = 0.50, *p* = 0.068) and IC-Si^B^ (*r* = 0.53, *p* = 0.054) against daily maximum currents. In contrast, residuals from the chl *a* versus time relationship did not show a correlation with maximum under-ice currents. It was only at a lag of one sampling cycle (3–4 days) that a slight (non-significant) negative correlation against daily maximum currents appeared (*r* = −0.37, *p* = 0.210).

## Discussion

Our study provides direct evidence for storage of intracellular (IC) nutrient pools by Arctic bottom-ice algal communities *in situ* at concentrations far exceeding that available in the under-ice water column. The averaged intracellular concentration and content estimates fell well within ranges of corresponding IC pools reported for both benthic and pelagic marine diatom taxa.^31^^,^^33^ Interestingly, intracellular pools were approximately double in 2012 relative to 2010, corresponding to a community-averaged cell volume that was approximately 1.5 times larger. The ice algal bloom observed in 2012 also had twice the chl *a* concentration. The sample site in 2012 was in a shallower location close to a tidally forced invisible polynya that occurs off the coast of Sheringham point (Figure 1). Similar invisible polynyas were shown to enhance ice algal blooms via increased under-ice mixing and sub-ice turbulence that increases ocean-ice nutrient fluxes.^9^^,^^42^ Therefore, although water column nutrient concentrations did not vary between sample years, the evidence of greater chl *a* accumulation and larger cells making up the ice algal community suggest the 2012 site likely experienced greater ocean-ice nutrient flux leading to greater nutrient uptake and subsequent IC nutrient pools. In the following, we discuss the implications of IC nutrient pools on the measurement of nutrient concentrations and availability in sea ice, before examining the potential role of IC nutrients in extending an ice algal bloom during spring.

### Measuring nutrients in sea ice

The existence of ice algal IC nutrient storage adds a significant complexity to our understanding of nutrient availability in the ice algal environment. It was originally suggested that IC nutrients could help explain the observed positive relationships of bulk ice nutrient versus chl *a* concentrations.^48^ An alternative mechanism was proposed by Roukaerts et al.,^49^ who hypothesized that a sea ice microbial biofilm could explain elevated nitrate concentrations in bulk sea ice measurements in the Antarctic via retention of heterotrophic remineralization products. However, we argue that such a mechanism would not contribute substantially to our intracellular measurements, as samples were collected from the permeable bottommost millimeters of the sea ice, where a brine channel clogging aspect of a biofilm^50^^,^^51^ would be minimal. We note our bulk ice NO_3_ + NO_2_ concentrations fell close to a 1:1 line with estimates of bottom-ice IC nutrient concentrations, suggesting a near complete release of IC NO_3_ + NO_2_ upon ice core melt processing. Therefore, measurements of bulk ice NO_3_ + NO_2_ largely represent the intracellular NO_3_ + NO_2_ pool of the ice algal community, confirming the previously stated hypothesis of Cota et al.^16^

If bulk ice NO_3_ + NO_2_ concentrations are representative of the intracellular pool, then a decrease in the slope to a lack of relationship between bulk ice NO_3_ + NO_2_ versus chl *a* concentrations could highlight different levels of N-limitation on ice algal growth. That is, if there is not enough NO_3_ + NO_2_ in the water column to sustain ice algal growth demands, then intracellular nutrient pools would not build up. For example, studies from the Kitikmeot Sea in the western Canadian Arctic report a lack of relationship between bulk ice NO_3_ + NO_2_ and chl *a*.^42^^,^^52^ As one of the most N-depleted systems of the Arctic Ocean,^53^ multiple lines of evidence have demonstrated ice algal growth to be N-limited in the region.^42^^,^^52^^,^^54^

In contrast to IC NO_3_ + NO_2_, IC Si(OH)_4_ was more than an order of magnitude greater than Si(OH)_4_ concentrations measured in the bulk ice melt. Diatoms are able to take up Si(OH)_4_ actively via specialized silicon transport proteins,^55^ or passively via diffusion across the cell membrane,^56^ and then store intracellular pools at concentrations that can exceed the saturation limit of 2 mM Si(OH)_4_.^31^ Although the intracellular soluble silicon transport mechanism is not known for diatoms,^32^^,^^57^ to keep IC Si(OH)_4_ concentrations above saturation levels, diatoms likely bind^31^ and, or stabilize the pool via production of specialized proteins.^55^ During asexual division, silicic acid is transported into specialized silicon deposition vesicles, where it is eventually deposited to form frustules.^57^ Here, we suggest that the complex specialization of diatoms to uptake, store, and internally transport intracellular silicic acid pools could act to resist its release due to osmotic stress during ice core melt processing for bulk nutrient measurements. If this suggestion holds, then bulk ice Si(OH)_4_ concentrations could strongly underestimate what is available to the ice algal cells. To place this statement in context, the IC Si(OH)_4_ content averaged 251 ± 134 fmol cell^−1^ in 2012, which compares to a bloom-averaged ice algal biogenic silica content of 920 ± 660 fmol cell^−1^ observed in Hudson Bay.^15^ Although the two studies are from different locations, this rough comparison highlights that the IC pool could likely support a single division or less. The suggestion that IC Si(OH)_4_ pools are not fully released during melt processing also helps explain the altered N:Si molar ratio in the bulk ice, which increased by nearly 4 times that measured in the under-ice water column. Therefore, bulk ice measurements of Si(OH)_4_ concentrations are unlikely to be representative of what is available to the ice algal cells.

### Temporal dynamics of intracellular nutrient storage

The tendency for IC-N^B^ to decrease over the seasonal transition, while IC-Si^B^ increased and the N:Si molar ratio decreased, suggests a decreasing intracellular pool of nitrogen relative to silicon. Many previous studies have concluded that nitrogen limits ice algal growth from the Pacific to the Canadian Arctic, as the high bottom-ice biomass accumulation typical of sea ice algal blooms is likely to rapidly increase demand over supply.^11^^,^^42^^,^^58^ Furthermore, silicon per cell can actually increase as growth rate decreases when Si is not limiting growth.^31^ In particular, using three separate centric diatoms, this increase was shown for diatom growth rate controlled by nitrogen limitation.^59^ Therefore, our data support the potential for onset of nitrogen limitation of the ice algal community as the bloom decreased overtime with no evidence of silicon limitation.

Within the overall seasonal trends, an oscillation was evident, corresponding to the spring-neap tidal cycle. Under the same mechanism described for the greater bloom extent observed in 2012, but over time instead of space, turbulent energy imparted by spring tides cause salinity isolines to shoal, the upper water column to mix, and the viscous boundary layer at the ocean-ice interface to thin, all of which act to enhance nutrient flux to the bottom-ice algal community.^11^^,^^41^^,^^60^ Turbulent currents also increase the delivery of nutrients to algal cells that are attached to the ice bottom versus that of phytoplankton in the water column.^42^^,^^43^^,^^60^ The positive correlations of temporally detrended intracellular nutrient concentrations against maximum current velocities suggest greater uptake during spring tides. This uptake appears to occur with a loss of algal biomass from the ice, followed by an increase in ice algal accumulation as neap tide approaches and intracellular stores decrease. Using photography of the ice bottom, Mundy et al.^61^ observed a similar mid-bloom sloughing event of the ice algal community in resolute passage. The ice algal community rapidly dropped from the ice matrix to “hang” in the water column while still anchored to the ice during neap tide. They speculated that nutrient limitation influenced the change in algal vertical location, while mechanical erosion of the hanging cells during spring tide occurred, followed by re-establishment of an internal bottom-ice community. Our data support this speculation, suggesting an oscillating replenishment of nutrients during spring tides as concluded previously.^11^^,^^41^ What is new is the potentially significant role of IC nutrient uptake as a mechanism to concentrate and retain nutrients during energetic spring tides, which we speculate help extend the ice algal bloom during the less energetic and limited nutrient supply neap tides.

### Conclusion

This field study directly documents that ice algae can internally store soluble nutrients at levels far exceeding those in their environment. This knowledge adds complexity to measurements of nutrient availability to ice algal communities where simple bulk melt techniques without filtered seawater addition seem only appropriate for determining soluble inorganic nitrogen, but not silicon. For silicon availability, intracellular measurements are best if possible; however, ice-water interface samples can provide more accurate information on nutrient availability to the ice algal community compared to bulk ice measurements. The ability to store intracellular nutrient pools likely assists ice algae in surviving harsh conditions of the sea ice environment, such as polar darkness, anoxia, or extended periods of limited nutrient supply. In particular, the mechanism to uptake and store intracellular nutrients is a beneficial adaptation for ice algae to extend blooms under a periodically enhanced ocean-ice nutrient supply. The mechanism also presumably plays a role in regulating buoyancy^62^ and thereby increasing the sinking rate of ice diatom cells. Furthermore, the capacity of ice algae to form aggregates and rapidly sink largely intact after bloom termination,^63^ represents a currently unquantified pathway of uptake and export of surface inorganic nutrients to the lower water column and benthos. Climate warming could exacerbate such a mechanism, provided the extreme example documented by Galindo et al.^13^ of an early rainfall-induced ice algal-seeded under-ice pennate diatom bloom that rapidly depleted upper water column NO_3_ + NO_2_ concentrations. Therefore, our documentation of intracellular nutrient accumulation by ice algae highlights an important biological process, not only for improving our understanding of algal ecophysiology and nutrient dynamics in the sea ice environment, but also with respect to implications for biogeochemical fluxes between pelagic and benthic environments in the Arctic marine system.

### Limitations of the study

Ice algae are inherently difficult to study as they reside within the sea ice matrix, a complex mixture medium of ice, brine, solid salts, and gas. Techniques to measure biogeochemical aspects of ice algae all involve removing the cells from their natural environment, which includes melting the ice so that the cells and environmental constituents can be filtered and processed. In this study, we attempted to minimize the impact of melt on the algal cells by scraping the cells into pre-filtered sea water (FSW), followed by filtering and filtrate collection as quickly as possible. Inevitably, the technique could still stress cells, which could in turn impact measurements of IC nutrient content and concentration. Furthermore, the scrape technique targets the bottommost millimeters of the sea ice algal community, versus the FSW-diluted and bulk ice melt techniques for particulate and nutrient concentration measurements, respectively, that target the bottommost centimetres of the sea ice. It should be noted that the ice algal community is strongly concentrated in the bottommost millimeters of the sea ice, justifying the original use of the scrape technique. A final limitation to consider was the use of chl *a* concentration to standardize between different techniques of scrape versus FSW-diluted ice melt versus bulk ice melt. Chlorophyll *a* can vary per cell dependent on acclimation strategies and response to resource limitations, and therefore, its application here could have introduced variability into the analyses.

## Resource availability

### Lead contact

Requests for further information and resources should be directed to and will be fulfilled by the lead contact, C.J. Mundy (cj.mundy@umanitoba.ca).

### Materials availability

This study did not generate new unique reagents.

### Data and code availability

• Data used in this paper are fully available at https://doi.org/10.34992/q15a-1e88.
• No code was used in this study.
• No other data are used in this study.

## Acknowledgments

Field support for the project was provided by the Polar Continental Shelf program of 10.13039/501100000159Natural Resources Canada and through Individual and Northern Research Supplement 10.13039/100002806Discovery grants from the 10.13039/501100000038Natural Sciences and Engineering Research Council of Canada to C.J.M., M.G., and M.L. We thank Sylvie Lessard for cell identification and enumeration used in this manuscript. Special thanks are extended to J. Ferland, M. Gale, M. Gourdal, J. Gagnon, G. Desmeules, and P. Guillot for logistical and data processing assistance. This is a contribution to the research programs of the Canada Excellence Research Chair unit at the 10.13039/100010318University of Manitoba, CEOS, ISMER, Québec-Océan, 10.13039/100014378Canadian Museum of Nature, and Polar Ocean Mitigation Potential (POMP) project funded by the 10.13039/501100000780European Union 10.13039/100018693Horizon Europe program (grant # 101136875).

## Author contributions

C.J.M. and M.G. conceived the research through discussion with E.L. C.J.M., M.G., V.G., M.L., M.P., and J.-E.T. contributed to data acquisition. C.J.M. conducted the data analysis with input from all co-authors. C.J.M. wrote the paper with substantial input from all co-authors.

## Declaration of interests

The authors declare no competing interests.

## STAR★Methods

### Key resources table

**Table:** 

REAGENT or RESOURCE	SOURCE	IDENTIFIER
**Deposited data**		

Intracellular nutrient manuscript dataset on CanWin Datahub	https://doi.org/10.34992/q15a-1e88	N/A

**Software and algorithms**		

Plots, Kruskal-Wallis test, and OLS regressions	SigmaPlot 12.5 software	N/A
SMA regressions	R version: 2023.12.0 + 369	N/A

### Method details

#### Field site and sample collection

All sampling took place on landfast first-year sea ice in Resolute Passage during the 2010 and 2012 Arctic-Ice Covered Ecosystem (Arctic-ICE) projects near Resolute Bay, Nunavut, Canada (Figure 1). Data were collected every 3–4 days between 8 May and 18 June, 2010 and every 4 days between 19 May and 8 June, 2012. Ocean currents were only available for 2010 only and were derived from a harmonic analysis of data acquired from a down-looking Acoustic Doppler Current Profiler, as presented in Mundy et al.^46^ Although both datasets are used to test the hypothesis of a dominant IC-nutrient pool mechanism by ice algae, the 2010 dataset is used in a time series analysis of ice algal IC-nutrient pools, while the 2012 dataset is used to compare common sea ice nutrient measurement techniques.

Snow depths were measured at every core extraction location, with targeted sampling of three different sites to capture the available range of snow depth conditions, including thin (<10 cm), medium (10–16 cm), and thick (>16 cm) snow covers. A transition to two different sample sites occurred in 2010, as sampling into late spring covered a change in surface conditions from snow cover to drained white ice and melt ponds.^46^ Bottom-ice samples were collected from each of these extraction locations using a Kovacs Mark II coring system (9-cm inner diameter) and processed for analysis of (i) bottom-ice chlorophyll *a* (chl *a*) concentration and community composition, (ii) intracellular nutrients and, (iii) bottom-ice bulk nutrients (2012 only).

For quantitative measurements of bottom-ice chl *a* and community composition, up to three ice cores were extracted from each site and the bottom 3 cm were pooled into isothermal containers before melt in 0.2-μm filtered seawater (FSW) to limit osmotic shock to the algae during melt processing.^19^ The FSW-diluted core solution was melted in the dark over a 15 to 20-h period. For intracellular nutrient measurements, a bottom-ice scrape sample was collected from 1 to 3 cores per sampling site depending on visible algal coloration. The scrape procedure used a stainless-steel knife to scrape off the soft skeletal bottom-ice layer, which contained the strongest coloration of algal matter (<0.5 cm), directly into 500 mL of FSW at a temperature near freezing.^22^ This technique minimizes stress on algal cells during ice melt processing by: (i) maintaining sample salinities similar to growth conditions at the ice-ocean interface, and (ii) reducing time of exposure to potentially stressful melt conditions, as all scrape samples were processed within 3 h of collection. For bulk ice nutrient measurements, the bottom 3 cm of an ice core was collected and placed immediately into a sterile bag (*Nasco Whirl-Pak*) and then melted in the dark over a 15 to 20-h period in the dark.

#### Sample processing

##### Salinity

Bulk salinity of the melted ice and salinity of the water column were measured with a hand-held conductivity meter (Cond 330i, WTW) and a Sea-Bird SBE 19plus V2 conductivity-temperature-depth (CTD) probe, respectively.

##### Bottom-ice chl a and community composition

Subsamples of the FSW-diluted core solution were filtered onto Whatman GF/F glass fiber filters (nominal pore size of 0.7 μm) for analysis of bottom-ice chl *a*. Filters were placed in 90% acetone for 18 to 24 h, and the extracted chl *a* was measured before and after acidification with 5% HCl using a 10-005R Turner Designs fluorometer. The concentration of chl *a* was then calculated using the equation described in Holm-Hansen et al.,^64^ and corrected for FSW-dilution to the melted volume of the bottom-ice core sections for estimates of mg chl *a* m^−3^ as well as sampled core area for estimates of mg chl *a* m^−2^. Subsamples from the FSW-diluted core solution were also preserved with acidic Lugol’s solution^65^ and stored in the dark at 4°C for later analysis of cell identification and enumeration. Cells >4 μm were identified to the lowest possible taxonomic rank using inverted microscopy according to Lund et al.^66^; however, information is only presented on total autotrophic cell abundance and percent contribution of dominant pennate diatoms. All measurements from the FSW-diluted samples were corrected for the dilution of FSW.

Biovolumes of diatom species were compiled from the data archive of Leblanc et al. (2012). When species biovolumes were not referenced, biovolumes were estimated according to cell shape and dimensions^67^^,^^68^^,^^69^^,^^70^^,^^71^^,^^72^^,^^73^^,^^74^ (M. Simard and M. Gosselin, unpublished data) using appropriate geometric formulas.^75^ Biovolumes of dinoflagellates and flagellates were compiled from the report of Olenina et al.^76^

##### Intracellular nutrients

The method used to extract the intracellular nutrient pool was adapted from.^28^ Within 3 h of collection, a subsample from the scrape sample was filtered onto a pre-combusted (450°C for 5 h) Whatman GF/F filter within an acid-cleaned filter head mounted on a large Erlenmeyer flask. Once enough material was concentrated on the filter (visible confirmation), vacuum pressure was released and a 60-mL acid-cleaned polyethylene tube, triple-rinsed with boiling reverse osmosis water, was suspended below the filtration head within the Erlenmeyer flask. Then, 40 mL of boiling reverse osmosis water was poured directly into the filter funnel. The water was left for 10 min, then vacuum pressure was restored and the filtrate was collected in the suspended tube. Following collection of the filtrate, the tube was sealed and placed immediately into the −20°C freezer. It was kept frozen until analysis of nitrate + nitrite (NO_3_ + NO_2_) and silicic acid (Si(OH)_4_) within 6 months using a Bran-Luebbe 3 autoanalyzer.^77^ Samples for Si(OH)_4_ determination were thawed for at least 24 h to minimize the issue of silicate polymerization when samples have been stored by freezing.^78^ Furthermore, dissolution of diatom opal frustules from the IC extraction technique was believed to be minimal due to the protective organic coating of live cell frustules,^79^ lower solubility of opal in freshwater versus seawater,^80^ and the limited 10-min exposure to hot water. Following the abovementioned protocol, a subsample of the boiling reverse osmosis water was also collected as a blank for every sample day. We limit results of this assessment to NO_3_ + NO_2_ and Si(OH)_4_ in the present study, as their concentration is often reported as a control of ice algal production (e.g.,^11^^,^^15^^,^^52^). Chl *a* measurements were also carried out on scrape samples using protocols described in the previous section. To compare IC nutrient concentrations from the scrape samples against bulk ice nutrient concentrations (Figure 3), IC nutrient concentrations were divided by the scrape-sample chl *a* concentration and then multiplied by the bottom-ice chl *a* concentration. This calculation provided a melt volume-corrected estimate of IC nutrient concentrations within the bottommost 3 cm of the sea ice. It is noted that these bottom-ice IC nutrient concentration estimates were independent of the bulk ice measurements described below. This melt volume-corrected estimate of bottom-ice IC nutrient concentration was divided by total community biovolume to obtain IC nutrient concentration per cell volume (mmol L^−1^) and by total community abundance to estimate IC nutrient cell content (fmol cell^−1^).

##### Bulk ice nutrients

For the 2012 bulk ice samples melted without the addition of filtered seawater, two subsamples of the bulk ice samples melted without the addition of filtered seawater were filtered through pre-combusted (450°C for 5 h) Whatman GF/F filters using a sterilized syringe. Filtrate was collected in acid-cleaned polyethylene tubes after three rinses with the filtrate, and stored at −20°C until analysis within 6 months. A coincident measurement of these nutrients was also determined for water collected at a 2-m water depth. Concentrations of NO_3_ + NO_2_ and Si(OH)_4_ were measured on the filtrate, as described above.

### Quantification and statistical analyses

For each cell identification and enumeration sample, at least 400 cells were enumerated over a minimum of three transects in a 10-mL settling chamber. Kruskal-Wallis tests for comparison of means with non-uniform distributions, Ordinary Least Squares linear regressions to examine relationships between variables, and Pearson’s correlation coefficients to examine association of two variables were calculated using SigmaPlot 12.5 software, while a Standardized Major Axis regression to examine the relationships between two independently estimated variables was accomplished using R version: 2023.12.0 + 369. *p* values less than 0.01 were considered statistically significant, apart from using 0.1 or less to identify “weak” correlations.
